# The effect of health quotient and time management skills on self-management behavior and glycemic control among individuals with type 2 diabetes mellitus

**DOI:** 10.3389/fpubh.2024.1295531

**Published:** 2024-04-03

**Authors:** Mengjie Chen, Man Liu, Ying Pu, Juan Wu, Mingjiao Zhang, Hongxia Tang, Laixi Kong, Maoting Guo, Kexue Zhu, Yuxiu Xie, Zhe Li, Bei Deng, Zhenzhen Xiong

**Affiliations:** ^1^School of Nursing, Chengdu Medical College, Chengdu, Sichuan, China; ^2^The Second Affiliated Hospital of Chengdu Medical College, China National Nuclear Corporation 416 Hospital, Chengdu, Sichuan, China; ^3^The First Affiliated Hospital of Chengdu Medical College, Chengdu, Sichuan, China; ^4^West China Second University Hospital, Sichuan University, Chengdu, Sichuan, China; ^5^Mental Health Center, West China Hospital, Sichuan University, Chengdu, Sichuan, China; ^6^Sichuan Clinical Medical Research Center for Mental Disorders, Chengdu, Sichuan, China

**Keywords:** type 2 diabetes mellitus, health quotient, time management, self-management, glycemic control

## Abstract

**Objective:**

The aim of this study was to evaluate the present status of self-management behavior and glycemic control in individuals diagnosed with Type 2 Diabetes Mellitus (T2D), as well as to examine the impact of health quotient (HQ) and time management skills on both self-management behavior and glycemic control.

**Methods:**

Between October 2022 and March 2023, a purposive sampling method had been utilized to select 215 participants with type T2D. The survey concluded a general information questionnaire, an HQ scale, a diabetes time management questionnaire and a self-management behavior questionnaire. The health quotient(HQ)encompasses the individuals’ knowledge, attitude toward health, and the ability to maintain their own well-being. The diabetes time management questionnaire was reverse-scored, with higher scores indicating an enhanced competence in time management. The path among variables was analyzed using structural equation modeling(SEM).

**Results:**

SEM showed that the direct effect of HQ on time management was −0.566 (*p* < 0.05), the direct effect of time management on the effect of self-management was −0.617 (*p* < 0.05), the direct effect of HQ on self-management was 0.156, and the indirect effect was 0.349 (*p* < 0.05); the relationship between health quotient and self-management was partially mediated by time management, with a mediating effect size of 68.8%. In addition, self-management had a direct effect on HbA_lc_, with a size of −0.394 (*p* < 0.05); The impacts of both HQ and time management on HbA_lc_ were found to be mediated by self-management, with HQ demonstrating an indirect effect of −0.199 (*p* < 0.05) and time management showing an indirect effect of 0.244 (*p* < 0.05).

**Conclusion:**

Health quotient and time management in patients with T2D serve as catalysts for self-management behavior. They affect HbA_lc_ level indirectly through self-management practices. The suggestion is to prioritize the cultivation of rational time organization and management skills in T2D patients, as well as enhance their health quotient level. This can facilitate a more effective improvement in patients’ self-management behaviors, ultimately achieving the objective of maintaining optimal glycemic control.

## 1 Introduction

Diabetes mellitus has emerged as an escalating global health crisis in the 21st century. According to the data disclosed by the International Diabetes Federation (IDF) in 2021, the prevalence of diabetes among the global adult population exceeded one-tenth (1). Within this context, China, as a densely populated nation, confronted a formidable challenge in the epidemic of diabetes (2). The prevalence rate of diabetes among Chinese adults was recorded at 11.2%, with type 2 diabetes mellitus (T2D) accounting for over 90% of the diabetes population (3). T2D is characterized by its notably elevated morbidity rates, a pronounced prevalence of disability, and the occurrence of severe complications in advanced stages. As a consequence, it profoundly impacts both the physical and psychological well-being of patients while imposing a substantial socio-economic burden.

The pivotal metric for assessing diabetes management remained glycated hemoglobin (HbA_1c_) (4). The current achievement of the recommended HbA_1c_ target of 7% among Chinese T2D patients was below 40% (5, 6). Diabetes self-management behavior was considered an important behavioral determinant in regulating blood glucose levels (5). The study demonstrated a positive association between diabetes self-management behaviors and effective glycemic control (7). However, the current state of self-management among patients affected by T2D remains suboptimal. The research findings suggested that only a range of 9.2 to 16.7% Chinese patients with T2D adhered to a regimen encompassing sufficient self-management behaviors (8).

Self-management behaviors are imperative for the regulation of diabetes as they exert a favorable impact on patients’ metabolism and psychological well-being. However, diabetes self-management necessitates a substantial time commitment. Diabetes care and education experts estimated that the performance of routine diabetes self-management might require a daily commitment of approximately 2 h, which could be further extended for patients who had recently diagnosed or possessed additional healthcare needs (9). Reaching a balance among self-management behaviors including dietary control, physical exercising and medication adherence played a crucial role in regulating blood glucose levels (10). Time management was found to be a predictor of glycemic control and adherence to diabetes management (10). Competence in diabetes time management was a significant and distinctive factor contributing to the explanation of self-care competence, with time management skills potentially influencing the successful implementation of self-care (11). Therefore, the assessment and training of skills related to scheduling constitutes an important area warranting further attention in academic research.

Health quotient was a comprehensive concept that encompassed an individual’s health awareness, knowledge and ability, as well as their wisdom, attitude toward health and ability to maintain it (12). Enhancing HQ can improve individuals’ recognition of risk factors associated with the occurrence and progression of chronic diseases, facilitate their acquisition of fundamental knowledge about chronic diseases, and foster a steadfast establishment of disease prevention concepts. By adopting this strategy, individuals are inclined to make accurate judgment and process their own health information in time, thereby establishing healthy behaviors and lifestyles, ultimately preventing the occurrence of chronic diseases at the root cause. By conducting an assessment of health literacy within the diabetic patient population, a comprehensive understanding can be achieved, with the aim of transforming patients’ cognitive framework and enhancing their health consciousness. This approach will effectively guide them toward developing self-management capabilities and adopting a healthy lifestyle (13), ultimately leading to optimized blood glucose levels.

In conclusion, the glycemic control among patients afflicted with T2D is suboptimal. The cornerstone of glycemic management in diabetes lies in effective self-management behaviors, with time management skills serves as the key for successful implementation of self-management behaviors. Concurrently, research probing the impact of HQ and time management on self-management among T2D patients is still in the early stage. Further study are needed to explore the action path on glycemic management and verify the relationship between these four factors. The objective of this study was to establish a structural equation model elucidating the relationship among HQ, time management, self-management and blood glucose level in patients with T2D. The model aims at exploring the action path of HQ and time management abilities on self-management behavior and blood glucose control, thereby enriching the theoretical framework for effective self-management behaviors and glycemic control in patients with T2D.

## 2 Methods

### 2.1 Design and participants

In this research, the subjects with T2D were selected from three tertiary hospitals in Chengdu, Sichuan Province using a purposive sampling method. Inclusion criteria: ① Age ≥ 18 years old; ② Patients meeting the diagnostic and classification criteria for T2D as stipulated by World Health Organization (14); ③ Requisite educational attainment at the elementary school level or higher; ④ Unobstructed awareness, adeptness in comprehension, and absence of hindrances to verbal communication.; ⑤ Absence of severe mental disorder, psychological dysfunctions, or cognitive impairments; ⑥ Informed consent and voluntary participation. Exclusion criteria: ① Patients in advanced stages of the disease (such as ketoacidosis, diabetic hyperosmolar coma, etc.), who exhibit an impaired ability to collaborate with the survey; ② Relevant coexistence of other severe chronic ailments (cardiovascular disorders, cerebrovascular incidents, malignancies, chronic pulmonary conditions, hepatic and renal disorders); ③ Individuals concurrently engaged in other interventional studies.

### 2.2 Instruments

1. General Information: A general information questionnaire was developed based on expert consultation, including age, gender, marital status, educational attainment, occupation, monthly household income, place of residence, and disease duration.
2. Health Quotient Scale (HQS): The Health Quotient Scale, developed by Guo et al. (15), Xie (16) based on the health concept, was utilized. The scale consisted of 100 items, encompassing five dimensions: self-care, health knowledge, lifestyle, mental state, and life skills. Each dimension was divided into four sections, each containing five items. Responses were evaluated on a 7-point continuum (ranging from 0 for “never” to 6 for “always”). HQ was categorized into four levels: 9–10 as “Optimal,” 6–8.99 as “Suboptimal,” 3–5.99 as “Warranting Caution,” and 0–2.99 as “Substandard.” (Calculation method: The total score of the five items was summed to determine the overall score for that section, which was then divided by three to derive the actual score. The sum of the actual scores for the four sections was divided by four to obtain the HQ index, which captured various dimensions of patients’ conditions. Similarly, the sum of the HQ indexes for the five dimensions was divided by five to determine the total HQ index of the surveyed subjects). The content validity coefficient of the questionnaire was 0.87, and the overall Cronbach’s coefficient was 0.91, indicating excellent questionnaire reliability and validity (17).
3. Diabetes Time-Management Questionnaire (DTMQ): The assessment of diabetic patients’ time management abilities was conducted using a questionnaire developed by Gafarian and Heiby (10). This questionnaire consisted of 49 items evaluated on a 5-point Likert scale, with higher scores indicating lower levels of time management proficiency. The questionnaire demonstrated good reliability with a Cronbach’s alpha coefficient of 0.82. In the current study, we obtained authorization from the original author to translate the DTMQ into Chinese and to validate the translation. This translation procedure followed the guidelines of Brislin’s classic back-translation model (18), encompassing both positive and negative translation phases. The Chinese version of the DTMQ encompassed 37 items, which were categorized into two dimensions: positive time management skills and negative time management skills. The former encompassed 32 positively scored items, while the latter involved five negatively scored items. Higher scores on the DTMQ were associated with decreased proficiency in time management skills. The questionnaire’s reliability and stability were underscored by a Cronbach’s alpha coefficient of 0.916, a test–retest reliability of 0.915, a split-half coefficient of 0.786, and a content validity of 0.976, which indicated a good reliability and stability.
4. Chinese version of Diabetes Self-Care Scale (DSCS): Wang et al. (19) conducted a translation and revision of the scale into Chinese in 1998, resulting in a total of 26 items distributed across six dimensions. These dimensions encompassed various aspects, including exercise self-care, dietary self-care, medication and blood glucose monitoring self-care, foot self-care, and prevention and management of high and low blood glucose. Employing the Likert 5-level scoring method, scores were calculated on a scale ranging from 1 (never) to 5 (always), yielding a cumulative scale value up to 130 points. Receiving lower scores was indicative of suboptimal self-management behavior. In this study, standardized scores were computed to categorize self-management behavior levels into high, moderate, and low grades (standardized score = actual score/possible highest score * 100). Final scores exceeding 80, scores ranging from 80 and 60, and scores below 60 represented high, moderate, and low levels, respectively. The scale demonstrated a Cronbach’s α coefficient value of 0.92 and had been widely applied among patients with type 2 diabetes (20).
5. Blood Glucose: In this study, the glycated hemoglobin (HbA_1c_) value was employed as an index for blood glucose assessment. Venous blood samples were collected from the patients and subsequently analyzed using High Performance Liquid Chromatography (HPLC). In accordance with the stipulations outlined in the guidelines of the American Diabetes Association (21), an HbA_1c_ level below 7% was adopted as a metric denoting optimal glycemic regulation.

### 2.3 Statistical analysis

Data were analyzed using SPSS 26.0 (IBM) and AMOS 26.0 software. Quantitative data were reported as mean ± standard deviation, and qualitative data were reported as n (%). Correlation among variables was tested using the Pearson correlation test, gender differences between variables were analyzed using independent samples t-tests. Structural equation modeling was performed using AMOS 26.0. The model was fitted using maximum likelihood estimation, which was adjusted based on the modification index. The significance level for testing was set at α = 0.05. The ideal criteria for model fit suggest that a *χ*^2^/df value less than 5 is acceptable, with values below 3 considered more favorable. Regarding absolute fit index, the root mean square error of approximation should be less than 0.08, while the adjusted goodness-of-fit index and the goodness-of-fit index should both be above 0.8 for an acceptable model fit, with values above 0.9 considered more favorable. As for relative fit index, the comparative fit index, normed fit index, and Tucker-Lewis index should all be above 0.8 for an acceptable model fit, with values above 0.9 considered more favorable.

### 2.4 Ethical considerations

The study was approved by the Ethics Committee of Chengdu Medical College (approval no. 2022–28). It was carried out in accordance with the Code of Ethics of the World Medical Association (Declaration of Helsinki). The study obtained informed consent from all participants.

## 3 Results

### 3.1 Patient characteristics

The study enrolled a total of 215 patients diagnosed with diabetes mellitus, aged between 22 and 83, with a mean age of (57.80 ± 12.10). Detailed information regarding other demographic characteristics can be found in Table 1.

**Table 1:** Sociodemographic characteristics of the study subjects (*N* = 215).

Characteristic	Category	n(%)
Gender	Male	129 (60)
	Female	86 (40)
Age, year	<60	127 (59.1)
	≥60	88 (40.9)
Education	Elementary School	47 (21.9)
	Middle School	90 (41.9)
	High School	57 (26.5)
	University and above	21 (9.8)
Intimate partner	Yes	186 (86.5)
	No	29 (13.5)
Type of employment	Government and institutions	16 (7.4)
	Company employee	38 (17.7)
	Farmer	19 (8.8)
	Freelance/Self-employed	46 (21.4)
	Retired	90 (41.9)
	Other	6 (2.8)
Location of residence	Urban	174 (80.9)
	Rural	41 (19.1)
Monthly *per capita* household income	<2000 RMB	46(21.4)
	2000–5,000 RMB	103 (47.9)
	5,001–10,000 RMB	51 (23.7)
	>10,000 RMB	15 (7)
Medical insurance	Self-funded	12 (5.6)
	Employee basic	142 (66)
	Urban and rural residents	61 (28.4)
Duration of diabetes	0–5 years	95 (44.2)
	6–10 years	41 (19.1)
	10 years or more	79 (36.7)

### 3.2 Present state of HbA_lc_

The HbAlc levels of 215 diabetic patients ranged from 5 to 18.2 mmol/L, with a mean value of (8.04 ± 2.10) mmol/L. Among them, HbAlc levels were below 7.0 mmol/L in 75 patients, achieving an attainment rate of 34.9%. In addition, HbAlc levels ranged between 7.0 and 10 mmol/L in 109 patients (50.7%), while exceeding the threshold of 10 mmol/L in 31 patients (14.4%).

### 3.3 Present state of HQ, time management, and self-management

**Figure 1:**
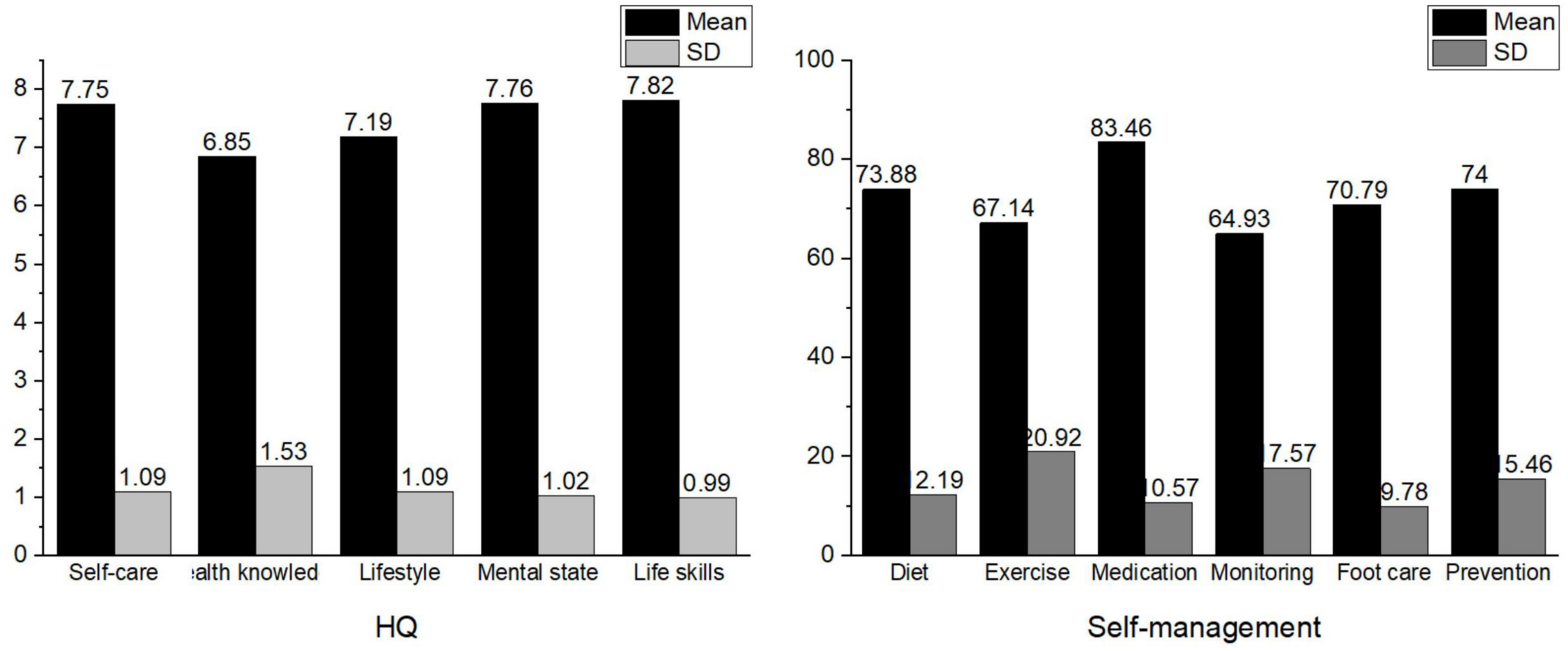
Scores on the dimensions of HQ and self-management.

In this study, the total HQ index for diabetic patients ranged from 4.68 to 9.90, with an average of 7.47 ± 0.97, indicating an generally favorable level. Notably, 14 individuals (6.5%) were at cautionary level; 186 individuals (86.5%) achieved a suboptimal level; and 15 individuals (7%) reached an optimal level. The scores for time management proficiency ranged from 49 to 139, with an average score of 89.07 ± 16.32. The standardized scores for self-management behaviors varied from 42 to 96%, with an average of 72.98% ± 10.06%, positioning the overall performance at a moderate level. Among these, 23 individuals (10.7%) demonstrated a low level of self-management, 151 individuals (70.2%) a moderate level, and 41 individuals (19.1%) a high level. The detailed scores for each dimension of the Health Quotient Scale (HQS) and the Diabetes Self-Care Scale (DSCS) are provided in Figure 1.

### 3.4 Gender differences in HQ, time management, self-management scores, and HbA_lc_

Independent samples t-test analyses revealed no significant gender differences in HQ, time management, self-management scores, or HbA_lc_ levels among diabetic patients, as evident in Table 2.

**Table 2:** Univariate analysis of the effect of gender on time management, HQ, self-management, and HbA_1c_ (*N* = 215).

Variable		$\bar{\chi }$ ± s	*t*	*p*
Time management	Male	88.27 ± 16.64	−0.87	0.384
	Female	90.26 ± 15.85		
Health quotient	Male	7.53 ± 0.97	1.03	0.302
	Female	7.39 ± 0.95		
Self-management	Male	72.12 ± 10.69	0.25	0.800
	Female	71.77 ± 9.09		
HbA_1c_	Male	8.03 ± 2.24	−0.15	0.878
	Female	8.07 ± 1.90		

### 3.5 The correlation among HQ, time management, self-management scores, and HbA_lc_

Pearson correlation analysis showed that HQ was negatively correlated with time management scores and HbA_lc_, and positively correlated with self-management; time management scores were strongly negatively correlated with self-management, and positively correlated with HbA_lc_; self-management scores were negatively correlated with HbA_lc_. The correlation matrix was detailed in Table 3.

**Table 3:** Pearson correlation analysis of HQ, time management, self-management, and HbA_lc_.

Variable	HQ	Time management	Self-management	HbA_lc_
HQ	1			
Time management	−0.520**	1		
Self-management	0.399**	−0.630**	1	
HbA_lc_	−0.231**	0.271**	−0.315**	1

**Significant at the 0.01 level (two-tailed).

### 3.6 Structural equation modeling of HQ, time management, self-management, and HbA_lc_

The research hypotheses guided the construction of an interaction model encompassing HQ, time management, self-management, and HbA_1c_. Time management, self-management and HbA_lc_ were the endogenous variables; HQ was the exogenous variable. Self-management was the endogenous latent variable. Which was assessed through measured variables including diet, exercise, medication, glucose monitoring, foot care. as well as strategies for preventing both high and low blood glucose levels.; HQ was the exogenous latent variable, which was evaluated by measured variables encompassing self-care, health knowledge, lifestyle, mental status and life skills.

The results indicated that *χ*^2^ = 145.099, df = 61, *χ*^2^/df = 2.379, and *p* < 0.001, indicating a failure to meet the fit requirements which was possibly due to an exceeding sample size of over 200. Consequently, evaluating the adequacy of the model’s suitability necessitated consideration of supplementary fitness indicators (6, 22). With model correction, guided by the modification indices, the final model fit was improved (23), as evident in Table 4.

**Table 4:** Fit of the modified model of interactions.

Fit criterion	Indicator	Standard value	Model fitted values	Accept the model?
Absolute fit index	GFI AGFI RMSEA	>0.9 >0.9 <0.08	0.908 0.863 0.080	Yes Yes Yes
Relative fit index	TLI CFI NFI	>0.9 >0.9 >0.9	0.915 0.934 0.892	Yes Yes Yes

AGFI, adjusted goodness-of-fit index; CFI, comparative fit index; GFI, goodness-of-fit index; NFI, normed fit index; RMSEA, root mean square error of approximation; TLI, Tucker-Lewis index.

**Figure 2:**
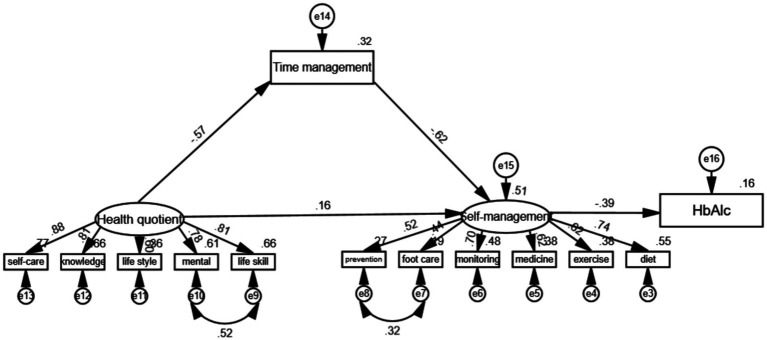
Structural equation modeling of HQ, time management, self-management, and HbA_lc_.

Figure 2 illustrates that HQ significantly impacts the time management path coefficient (*β* = −0.57, *p* < 0.05), and time management significantly affects the self-management path coefficient (*β* = −0.62, *p* < 0.05), signifying a notable mediating effect of time management between HQ and self-management, quantified by the mediating effect a × b = (−0.57) × (−0.62) = 0.3534. After accounting for the mediation effect of time management, the coefficient of HQ on the self-management pathway remained significant (*β* = 0.16, *p* < 0.05), suggesting a partial mediating role of time management between HQ and self-management. The proportion of mediating effect to total effect was calculated as ab/c = 0.688(i.e., 68.8% of the total effect size). Furthermore, the self-management path coefficient on HbA_1c_ was significant (β = −0.39, *p* < 0.05). Both HQ and time management indirectly influenced HbA_1c_ through self-management, generating respective indirect effects of −0.199 and 0.244. The supplementary path coefficients are outlined in Table 5.

**Table 5:** Path analysis of the effect of HQ, time management on self-management, and HbA_lc_.

Path	Direct effect	Indirect effect	Total effect
HQ → Time management	−0.566	—	−0.566
HQ → Self-management	0.156	0.349	0.505
HQ → HbA_lc_	—	−0.199	−0.199
Time management → Self-management	−0.617	—	−0.617
Time management → HbA_lc_	—	0.244	0.244
Self-management → HbA_lc_	−0.394	—	−0.394

## 4 Discussion

The health quotient, time management skills, self-management behaviors, and HbA_1c_ levels among type 2 diabetic patients showed no significant gender differences. The studies conducted by Yao et al. (24) and Hong et al. (25) both revealed no gender differences in the level of HQ among diabetic patients, corroborating the findings of the present study. Furthermore, it was discovered (26) that an individual’s time management ability was less influenced by gender, although studies exploring the impact of gender on time management ability in diabetic populations have not been reported. Caruso et al. (27) observed no significant gender difference in self-management. Yet in a subsequent study (27), they found that women’s self-management ability was comparable to or superior to men’s. Wang et al. (28) reported that women had lower HbA_1c_ levels than men, whereas Hoffman et al. (29) found no gender difference in HbA_1c_ levels, consistent with the results of the present study. Gender differences in self-management behaviors and HbA_1_c levels in diabetic patients may be influenced by factors such as geographic and cultural disparities, rendering it challenging to reach consistent conclusions. Further investigation through extensive and protracted studies is imperative to reach a more comprehensive understanding.

### 4.1 Effect of self-management on blood glucose

The compliance rate for glycemic control among patients with T2D in this study was found to be 34.9%, indicating suboptimal glycemic management, which was consistent with the findings reported by Lin et al. (5). Glycemic control stood as the cornerstone of diabetes management, with evidence linking elevated blood glucose levels intricately to cardiovascular and microvascular complications (30, 31). Thus, a comprehensive study of the factors influencing blood glucose levels assumes paramount significance. This study revealed a significant negative correlation between self-management behaviors of diabetic patients and HbA_1c_ values. Additionally, the outcomes of the structural equation modeling underscored a direct effect of self-management behaviors on blood glucose control, with an effect size of −0.394. This signifies that higher levels of self-management was associated with lower HbA_1c_ levels, indicating improved glycemic control in patients. Numerous studies (5, 32, 33) have shown that self-management behaviors were an integral part of optimizing glycemic control and preventing disease progression and related complications.

Self-management encompasses a spectrum of six primary behaviors: dietary control, physical activity, pharmaceutical adherence, glucose monitoring, foot care, and prevention of hyperglycemic and hypoglycemic events. The investigation revealed that dietary self-management stood as a robust predictive determinant of patients’ glycemic control (34). Concurrently, the American Diabetes Association (35) stipulated that achieving nearly normal or normal blood glucose levels mandated comprehensive self-management education coupled with intensified treatment strategies, which encompassed regular physical exercise and self-monitoring of blood glucose. Regular physical exercise could attenuate the onset and progression of diabetes and its associated complications (36). Research indicated that individuals with diabetes could achieve optimal blood glucose levels through the adoption of healthful dietary plans, routine physical activity, and weight management (37, 38). Consistent self-monitoring of blood glucose levels assumed pivotal importance in achieving and sustaining patients’ glycemic targets, facilitating assessments of glycemic status, prescribing optimal therapeutic regimens, and ensuring prompt treatment adjustments (39, 40). This significance was particularly pronounced among patients undergoing insulin therapy (41). Studies have demonstrated (42, 43) a positive correlation between higher rates of self-monitoring on blood glucose, especially when performed eight times or more daily, and enhancements in glycemic control. Furthermore, the impact of patients’ self-management of medication on glycemic control is of paramount importance. Evidence underscored (44) that inadequate adherence to diabetes medications could compromise glycemic control, thereby exacerbating diabetes progression, precipitating complications, increasing hospitalization rates, and elevating mortality rates (45). Regular foot care and examinations for diabetes patients could effectively mitigate the occurrence of diabetic foot complications, reducing the risk of lower extremity amputations (46, 47). Additionally, the prevention of hyperglycemia and hypoglycemia demands attention. Elevated blood glucose levels was independently linked to microvascular complications while episodes of hypoglycemia exhibited a significant correlation with preclinical atherosclerosis (48).

These findings underscore the importance for healthcare practitioners to impart comprehensive self-care plans to patients during their health education sessions. This entails not only emphasizing adherence to timely and regulated medication intake but also devising personalized self-monitoring strategies for glucose levels and providing guidance on appropriate dietary habits and physical activities for individuals with diabetes. Equally crucial is providing patients with comprehensive guidance on timely management strategies in the event of hyperglycemic and hypoglycemic episodes.

### 4.2 Effect of HQ on self-management and blood glucose

This study revealed a significant positive correlation between HQ and self-management. The level of HQ in diabetic patients directly influenced their self-management, with an effect size of 0.156. This signifies that an increased diabetes HQ index corresponds to elevated levels of self-management. Furthermore, HQ exerted an indirect influence on HbA_1c_ values through self-management, with an indirect effect of −0.199. As the HQ index expanded, accompanied by heightened self-management levels, a consequential reduction in HbA_1c_ values ensued, thereby fostering enhanced blood glucose control. HQ encapsulates an individual’s orientation toward health and the echelon of their self-management concerning health. This includes multifarious dimensions encompassing the physical, psychological, emotional, cognitive, socio-environmental, and quality-of-life domains. Assessing HQ levels empowered individuals to recognize their health deficiencies, refine personal health perceptions, and establish wholesome living patterns (25). Research revealed (49) that integrating the concept of HQ into self-educational management among community-based diabetes patients could aptly guide a shift in patients’ health attitudes, catalyzing the enhancement of self-awareness, health knowledge, and health aptitude, consequently ameliorating patients’ self-management proficiency. A higher HQ could enhance patients’ full understanding of disease-related knowledge, in conjunction with a positive mindset, effective lifestyle choices, and judicious medication usage. This collective approach had the potential to yield superior outcomes in glycemic control, thereby facilitating adherence to glycemic targets (24). Furthermore, health education founded upon HQ principles fostered the cultivation of health behavioral competence within Type 2 diabetes patients, heightening blood glucose control standards, and augmenting patients’ satisfaction with health education (50).

Healthcare professionals and community workers should give appropriate attention to the HQ levels of patients. This encompasses imparting patients with self-care techniques such as accurate blood glucose monitoring and selection of suitable exercise modalities. Furthermore, conducting regular health seminars can enhance patients’ awareness of disease-related health knowledge, thereby guiding them toward adopting healthier lifestyles. Additionally, the psychological well-being of patients should not be disregarded. Timely and relevant provision of psychological solace and counseling is imperative to promote their life skills. Cultivating patients’ HQ index can foster robust self-management proficiency, ultimately facilitating a more effective blood glucose management.

### 4.3 Effect of time management on self-management and blood glucose

The study revealed a statistically significant negative correlation between the time management score and the level of self-management behavior, along with a positive correlation with the HbA_1c_ values. Concurrently, the time management score of the diabetic patients exhibited a direct influence on the level of self-management behavior, with an effect size of −0.617. Lisa et al. (11) found a significant strong negative correlation between diabetes self-care and time management with a correlation coefficient of −0.605, which is similar to the results of this study. This implies a positive correlation between the patient’s time management skills and their level of self-management behaviors. Furthermore, the indirect impact of time management competency on blood glucose levels was mediated through self-management behaviors, manifested by an indirect effect size of 0.244. Zhou (51) found that time management was significantly associated with improvements in patients’ blood glucose levels. This underscores that heightened time management competency contributes to reduced HbA_lc_ values through the facilitation of heightened self-management behaviors, culminating in the achievement of favorable glycemic control.

An analogous observation reported in an American study (11), where it was identified that time management among female individuals with T2D played a distinct role in promoting more robust and statistically significant self-management behaviors. Further reinforcing this, Jones (52) discerned that a patient’s time management acumen exerted a great influence on their adherence to self-management practices. In particular, individuals with diabetes who maintained consistent schedules and pursued an active lifestyle (53), along with those who occupied managerial roles in their professional sphere (54), showcased higher adherence to self-management protocols. These individuals exhibited superior time management and goal-setting competencies, allowing them to align their daily routines with desired health outcomes, thus augmenting their capacity to enhance glycemic control.

While patient education has been the primary focus of numerous interventions, there has been a tendency to overlook the crucial role of time management in diabetes self-management. Consequently, a pivotal domain within diabetes self-management behaviors, warranting heightened consideration, pertains to the assessment and cultivation of patients’ adeptness in temporal governance. Diabetes educators should promptly assess and enhance time management skills in diabetic patients. Time management ability is the guarantee of successful self-management behaviors in diabetic patients. Therefore the cultivation of time management ability can be started from self-management behaviors such as diet, exercise and medication taking. Regarding diet, patients can be advised to adopt time-restricted eating. Time-restricted eating has been found to reduce fasting insulin, improve glucose tolerance and reduce glycemic excursions (55, 56). Optimizing the administration of medication according to biological rhythms, taking medication at the optimal time period for drug efficacy, and conducting blood glucose monitoring after medication can reduce the occurrence of hyperglycemia or hypoglycemia. As for exercise, patients can be suggested to conduct limb function training or exercise during the peak performance of the human body. Studies have indicated (57) that exercising 120 min after a meal had the best effect on glycemic improvement, and hypoglycemia was less likely to occur. Patients should be instructed to maintain a reasonable duration of sleep. Research has shown that (58, 59) patients with habitually short sleep duration (<4.5–6 h per night) and prolonged sleep (>8–9 h per night) exhibited higher HbA1c levels compared with normal sleep duration.

### 4.4 The mediating role of time management

This study also found that time management partially mediated the relationship between HQ and self-management behaviors, with a mediating effect size of 68.8%, which meant that HQ levels of diabetic patients can influence their self-management behaviors through the mediation of time management skills. The Information-Motivation-Behavioral Skills model (60) posited that knowledge underpinned behavioral transformation with behavioral skills functioned as the direct facilitators of behavioral alteration. Knowledge assumed the role of a catalyst in enhancing behavioral skills, thereby orchestrating behavioral change. Furthermore, individuals endowed with elevated behavioral skills exhibited an augmented propensity to instigate behavioral modification and sustained such alterations over time. HQ served as a knowledge variable, while self-management behavior functioned as a behavioral outcome. Time management skills encompassed a set of behavioral skills including the formulation and adherence to plans, the structuring of a daily regimen, autonomous temporal regulation, prioritization of tasks, intricate problem-solving, and the segmentation of tasks into discrete components (61). On one facet, the HQ could directly contribute to the enhancement of self-management behaviors among diabetic patients. Simultaneously, HQ could indirectly catalyze heightened self-management behavior levels in patients by facilitating the refinement of their time management skills. Both of these dimensions invariably culminated in the augmentation of glycemic control. Notably, it was ascertained that elements encompassing self-care, lifestyle (62), health knowledge (54), and mental well-being (11) within the HQ construct could significantly impact the time management skills of diabetic patients. Patients who adhered to a healthy lifestyle (62) and engaged in diabetes education initiatives (54) tended to allocate more time toward self-management endeavors.

The cultivation of time management ability can elevate the cognitive echelons of health empowerment among diabetic patients, thereby enabling them to exhibit a proactive subjective stance toward effectual self-monitoring, self-management, and self-enhancement (63). As such, the influence of HQ on the self-management conduct of diabetic patients can be magnified through the cultivation of their temporal management aptitude. Healthcare practitioners can guide patients to self-administer based on the individual’s biorhythms, ensuring that these self-management behaviors are synchronized with the body’s insulin secretion patterns and inherent temporal biorhythms. By fostering patients’ abilities to schedule activities aligned with diabetes management within temporal frameworks, this has the potential to enhance the level of self-management behaviors of the patients, thereby presenting an opportunity for a novel clinical approach to improve glycemic control among them.

## 5 Limitations

The present study was a cross-sectional study, limiting the ability to establish causal relationship among variables. Additionally, although the sample was representative, there is potential for further expansion. The longitudinal study approach can be used in future studies to further investigate the pathways of HQ and time management ability of diabetic patients on self-management behaviors and blood glucose levels, thereby validating and expanding upon our findings.

## 6 Conclusion

The findings of this study have unveiled, for the first time, that HQ and time management were facilitators in enhancing the level of self-management among individuals with T2D. Consequently, these factors indirectly impact patients’ glycemic control through their influence on self-management practices. The relationship between HQ and self-management was partially moderated by time management. The influence of HQ, time management and self-management on glycemic control must not be disregarded. Diabetes educators should acknowledge the significant indirect effects of HQ and time management on glycemic control, while prioritizing patients’ self-management behaviors.

## Data availability statement

The raw data supporting the conclusions of this article will be made available by the authors, without undue reservation.

## Ethics statement

The studies involving humans were approved by Ethics Committee of Chengdu Medical College. The studies were conducted in accordance with the local legislation and institutional requirements. The participants provided their written informed consent to participate in this study.

## Author contributions

MC: Conceptualization, Data curation, Investigation, Methodology, Software, Writing – original draft. ML: Conceptualization, Investigation, Resources, Writing – original draft. YP: Investigation, Project administration, Writing – original draft. JW: Investigation, Project administration, Writing – original draft. MZ: Data curation, Investigation, Writing – original draft. HT: Data curation, Investigation, Writing – original draft. LK: Data curation, Investigation, Writing – original draft. MG: Investigation, Methodology, Writing – original draft. KZ: Investigation, Writing – original draft. YX: Investigation, Writing – original draft. ZL: Conceptualization, Supervision, Validation, Writing – review & editing. BD: Conceptualization, Supervision, Validation, Writing – review & editing. ZX: Conceptualization, Methodology, Project administration, Resources, Supervision, Writing – review & editing.
