# Supplementary material for: The effect of health quotient and time management skills on self-management behavior and glycemic control among individuals with type 2 diabetes mellitus
Source: Front Public Health. 2024 Apr 3;12:1295531. doi: 10.3389/fpubh.2024.1295531 (PMC11021650; doi:10.3389/fpubh.2024.1295531)
Supplement: Supplementary file 1 [file Data_Sheet_1.PDF]

Numbering:_______

Effects of Fitness Quotient and Time Management Skills on Self-Management Behavior and Blood Glucose in Type 2 Diabetes Mellitus Patients

Please evaluate the following before conducting the formal survey, and only proceed with the follow-up survey if you answer yes to all of the questions.

1. Whether diagnosed with diabetes mellitus; ①No□ ②Yes□
2. Consciousness, ability to read, no language communication barriers; ① No□ ② Yes□

Name of investigator:_____________

Date :_____________

Notice

Dear Diabetic:

Hello!

I am Mengjie Chen, a graduate nursing student at Chengdu Medical College, and I am currently conducting a study on the effects of health quotient and time management ability on self-management behaviors and blood glucose in patients with type 2 diabetes mellitus. You are invited to participate in this study because you are eligible for enrollment.

The purpose of this study is to construct a structural equation model to provide a richer and more accurate theoretical basis for improving the fitness quotient, time management ability and self-management behavior of diabetic patients by understanding the current situation of fitness quotient and time management of type 2 diabetic patients, as well as their influencing factors and exploring the effects on self-management behavior and blood glucose.

We need you to answer the following questions truthfully. This set of questionnaires consists of 4 sets of questionnaires, namely: "1. Basic Information Questionnaire", "2. Health Quotient Scale", "3. Diabetes Time-Management Questionnaire", and "4. Chinese version of Diabetes Self-Care Scale".

Please read the corresponding table in turn, and put a "√" in the "□" or fill in the accurate information in the space of the choice item that best meets your actual situation. This questionnaire is filled out anonymously, your information will not be disclosed to anyone, and the survey data will only be used for scientific research. Thank you for your support!

Researcher Contact:
Mengjie Chen, Tel: 13032823518, Email: ccmenjie@163.com

I have been made aware of the research study and have volunteered to participate in the program.

Signature:___________

Contact Information:_____________

Date:______

I. general information

1. Gender: ① Male☐ ② Female☐

2. Age:____

3. Education: ① Elementary school☐; ② Middle School☐; ③ High school☐; ④ University and above☐

4. Marital status: ① Married☐; ② Unmarried☐; ③ Divorced☐; ④Widowed☐

5. Type of employment: ① Government and Institutionalized employee☐;②Company employee☐; ③ Farmers ④ Freelance/Self-employed☐;⑤ Retired☐;⑥ Other (specify)__________

6. Location of residence: ① Urban☐ ; ② Rural☐

7. Per capita monthly household income: ① ＜2,000RMB☐ ; ② 2001-5000 RMB☐ ; ① 5001-10000 RMB 5001-10000RMB☐ ; ④ ＞10,000RMB☐

8. Ways of bearing medical expenses (Multiple choices allowed): ① Self-funded☐ ; ② Employee Basic Medical Insurance☐ ; ③ Basic Medical Insurance for Urban and Rural Residents☐ ; (iv) Publicly-funded medical care☐ ; ⑤ Commercial insurance☐ ; ⑥ Other_____________

9. Duration of diabetes: 0-5 years☐; 6-10 years☐; ＞10 years☐.

II. Health Quotient Scale

The following is a survey about your health quotient for disease, divided into 5 sections: self-care, health knowledge, lifestyle, mental status and life skills.

(1) self-care

Instructions for filling in the form: Please check "√" the one you agree with most among the 7 options (fully agree - fully disagree).

entry	totally agree	agree with	Kind of agree	neutral	Kind of disagree	disagree	Completely disagree
1. Self-care is important for physical and mental health							
2. I am willing to try new ways to maintain my health when practicing health care							

3. I prevent diseases caused by improper lifestyle through self-care							
4. I regularly practice self-relaxation and stress reduction techniques							
5. I control my weight, do self-examinations, be alert to early signs of illness and take preventive measures							
6. I can actively make decisions that meet my self-care needs							
7. I feel comfortable taking care of myself when I have a minor illness or infection							
8. I am satisfied with the health care staff							
9. Psychological effects are important during illnesses							
10. I am satisfied with my ability to choose a different approach to healthcare							
11. I enjoy good health							
12. I am satisfied with my mental condition							
13. I often feel pain and discomfort							
14. I can determine what certain physical symptoms mean							
15. I can recognize my own stress and emotional disturbances							
16. I have the ability to meet deadlines for what needs to be done							

17. I can prioritize my work properly							
18. Feeling relaxed, happy and cheerful with others							
19. I can see things in a logical perspective							
20. I have a good life.							

(2) health knowledge

Instructions for filling in the form: Please check "√" the one you agree with most among the 7 options (fully agree - fully disagree).

entry	totally agree	agree with	Kind of agree	neutral	Kind of disagree	disagree	Completely disagree
1. I know a lot about the disease							
2. I am satisfied that my knowledge of health is sufficient to assess my own health							
3. Mental health is important to overall health							
4. It is important to participate in regular physical exercise, eat a balanced diet, live regularly, and combine work and rest							
5. Healthy means more than just being disease-free							
6. I am knowledgeable about the current health care system							
7. I apply the health care system							

appropriately							
8. I have a greater knowledge of the various options for health care							
9. I am confident in my ability to choose the right health care system							
10. I would like to find more alternative health care methods to use							
11. I have been using an integrated holistic approach to my health, and I want to keep up to date with the latest advances in certain areas of medicine							
12. I make careful use of health information materials							
13. I pay close attention to the latest health technologies or methods							
14. When my behaviors and lifestyle affect my health, I can know that							
15. I keep up to date with the latest disease screening methods							
16. I know a lot of risk factors that contribute to disease							
17. I am familiar with the potential hazards of some							

over-the-counter drugs and supplements							
18. I receive frequent physical examinations, including screening for risk factors							
19. I take care to monitor my health with the right health information							
20. I have a good knowledge of the various environments that are harmful to my health							

(3) lifestyle

Instructions for filling in the form: Please check "√" the one you agree with the most among the 7 options (fully agree - fully disagree).

entry	totally agree	agree with	Kind of agree	neutral	Kind of disagree	disagree	Completely disagree
1. I do not smoke cigarettes, cigars, or pipes, nor do I otherwise smoke							
2. I avoid indirect smoking							
3. I drink a lot of alcohol every day							
4. I don't use alcohol or drugs to de-stress							
5. I pay attention to label directions when using prescription or over-the-counter medications							

6. I eat all kinds of food							
7. I care about what I eat.							
8. I think vitamins are important							
9. I eat regularly							
10. I'm very calorie-conscious.							
11. I'm concerned about my weight							
12. Appropriate physical activity, such as walking, is important for health							
13. I realized that rigorous exercise increases muscle elasticity and strengthens the heart							
14. I regularly participate in family or group activities that strengthen my body and mind							
15. I feel that I participate in enough physical activities and exercise to keep me strong and healthy							
16. I can cope with the stress of daily work and activities							
17. I am satisfied with my ability to perform daily activities							
18. I am satisfied with my ability to do my job							
19. I am satisfied with my sex life							

20. I feel that I live in a safe and secure environment							

(4) mental state

Instructions for filling in the form: Please check "√" the one you agree with most among the 7 options (fully agree - fully disagree).

entry	totally agree	agree with	Kind of agree	neutral	Kind of disagree	disagree	Completely disagree
1. I am confident about life and the future							
2. I am satisfied with my ability to think correctly, to enjoy learning and reasoning, and to pay full attention to myself							
3. My self-esteem is important to my health and well-being							
4. I am satisfied with my appearance and looks							
5. I am satisfied with my overall psychological condition, such as positivity, empathy and zest for life							
6. I can realize when my emotions are abnormal							
7. Anger, anxiety, and repressed emotions can be detrimental to health							
8. I can cope with stress and have more ways of coping when under pressure							

9. I like to socialize							
10. Controlling emotions is important for maintaining physical health							
11. Personal faith adds meaning to my life							
12. Personal faith helps me to overcome difficulties in life							
13. Personal faith makes me happier and happier							
14. Personal beliefs maintain my health, such as helping me to overcome illness, sadness, despair and helplessness, and helping me to maintain a healthy lifestyle							
15. Personal spirituality makes me feel healthy							
16. I like to do my own work (study)							
17. I can express my feelings easily and freely							
18. I can detect potential stress							
19. I have relatives and friends I can talk to when I need help or have something to talk to someone about							
20. I am capable of dealing with stress							

(5) life skill

Instructions for filling in the form: Please check "√" the one you agree with most among the 7 options (fully agree - fully disagree).

entry	totally agree	agree with	Kind of agree	neutral	Kind of disagree	disagree	Completely disagree
1. I have more skills in making decisions about my health and addressing it							
2. I used creative thinking to solve the problem							
3. I am satisfied with my skills in communicating and interacting with people							
4. I strive to be empathetic and compassionate							
5. I can handle emotions and stress							
6. I can balance work and family life well							
7. My financial resources enable me to live a full and happy life							
8. I have a high level of career satisfaction							
9. My work (school life) atmosphere enables me to express myself well, get along well with people, and accomplish daily tasks							
10. I live in a beautiful							

environment							
11. I can organize my life							
12. I can resist bad influences from my peers, such as drug or alcohol abuse							
13. I participate in social and recreational activities and enjoy them							
14. I am satisfied with my ability to obtain outside support							
15. I try not to be withdrawn and self-absorbed							
16. I feel alone							
17. I feel that I have good relationships with family members							
18. I feel I have a good relationship with non-family members							
19. I am satisfied with the support and dedication I give to others							
20. I am satisfied with the support I receive from family, friends and others							

III. Diabetes time management questionnaire

The following is a survey about your diabetes time management skills. Instructions for completion: Please tick one of the 5 options (All time - Never) with which you most agree.

entry	All time	most of the time	Sometimes	Very little time	Never
1.I have enough time to accomplish my daily responsibilities					
2.I use my time effectively and efficiently					
3.I am focused on my time management					
4.I wake up and go to sleep at approximately the same time every day and night					
5.I use my diabetes medication (insulin or pills) at about the same time each day					
6.I complete tasks on time (eg, paying bills on time, showing up for appointment on time)					
7.I eat my meals at about the same time each day					
8.I schedule at least 3 workouts per week					
9.I usually complete my blood glucose monitoring at the set time					
10.When I have more to do than I can accomplish in a day, I will give priority to the most important things					
11.I keep my diabetes healthcare plan on a regular schedule					
12.I will take my diabetes medication, such as insulin or pills, at the correct time					
13.When I have a lot things to do, I like to complete the hardest tasks first					
14.I exercise at about the same time every week					
15.I make lists of things I have to do every day					
16.I make a list of the things I have to accomplish eventually					
17.I check my blood glucose approximately 30 to 45 minutes prior to eating					
18.When my plan is interrupted by something unexpected, I will reschedule another time to do what I planned to do					
19.I accomplish the set tasks every day					
20.I set specific goals (e.g., I will jog 3 miles at 7:00 a.m. every morning) rather than vague goals (e.g., I will start exercising more frequently)					
21.I set reasonable goals (e.g.,on the first day of a new					

exercise plan, jogging 1/2 mile instead of 3 miles)					
22.When a task seems too large or difficult , I I break it up					
23.I am able to find a balance between both work and leisure time					
24.I know where I spend my time					
25.I set goals to improve my diabetes control (e.g., losing 5% of body weight in 3 months; lowering glycated hemoglobin by 1% every 3 months)					
26.I keep my diabetes supplies (blood testing stripe and blood glucose meter,syringes,etc) in an organized place where I can always find them					
27.When I don’t accomplish my goals,I know the reason why					
28.When unexpected changes occur in my schedule which may affect my diabetes control ,I am able to make quick decision about modifying my diabetes regimen(eg, adjusting the amount of medication you take or the amount food you eat.)					
29.I feel good when I finish a task					
30.I have a plan before the day starts					
31.I keep a record of my blood glucose values					
32.There is room for improvement in the way I manage my time					
33.I put off doing the things I wanted to do (even though I had time)					
34.I feel overwhelmed by what I need to do in a day					
35.I have procrastination					
36.I can't control my time					
37.I find myself rushing to get things done at the last minute					

IV. Chinese version of Diabetes Self-Care Scale

The following are statements about your implementation of diabetes self-management behaviors.

Instructions for completion: Please tick one of the 5 options (never - all the time) with which you most agree.

	Self-management behaviors	Never	Very little time	Sometimes	Most of the time	All the time
Diet (6)	1. I eat on time every day					
	2. I follow the principles of the diabetic diet at meals					
	3. I still follow the diabetic diet when I go to a friend's or relative's house for a meal					
	4. I eat with people I don't know well and still follow the principles of the diabetic diet					
	5. I still follow the principles of the diabetic diet on holidays, birthdays or when I go out to socialize					
	6. I make equivalent exchanges in the same food group					
Movement (4)	7. I usually exercise consistently (30-60 minutes more than 3 times per week)					
	8. when I subjectively don't want to exercise, I still keep exercising to control my blood glucose					
	9. Even if I'm busy, I make time to exercise					
	10. I make time to exercise on weekends or holidays					
Taking medication (3)	11. I usually take my medication at the time prescribed by my medical staff					
	12. I always take my medication at the dose prescribed by my medical staff					
	13. I still manage to take my diabetes medication on time when I am away from home					
Blood glucose monitoring (4)	14. I usually do regular blood glucose monitoring					
	15. I still monitor my blood glucose regularly when I am away from home					
	16. I recorded my blood glucose values each time					
	17. I test my blood glucose more often when I don't feel well					
foot care (5)	18. I usually wear soft-soled, loose-fitting, breathable shoes and socks					
	19. I still wear soft-soled, loose-fitting, breathable shoes and socks when I go out to socialize					
	20. I check my feet daily and apply moisturizer on them					
	21. I had my nails built as recommended by the medical staff					

	22. When there is an abnormality on the foot (e.g., skin cracks and abrasions, blisters, redness, swelling, corns, pain), I immediately seek medical treatment					
Prevention of high and low blood glucose (4)	23.When blood glucose control is poor (e.g., consistently too high or too low), I will seek help from a medical professional					
	24. When I feel that my blood glucose is too low (e.g., when I have a cold sweat, weakness, dizziness) I immediately use the correct treatment (e.g., drinking sugar water, eating candy, cookies)					
	25. When my blood glucose is higher than usual (e.g., when I feel thirsty, urinate excessively, or feel nauseous), I immediately take the correct self-management measures (e.g., drinking more water, adjusting my diet) and consult my doctor to adjust my medication					
	26. When exercising, I take steps to prevent hypoglycemic reactions (e.g., not exercising on an empty stomach, carrying candy or cookies with me when exercising)					

Raw Data

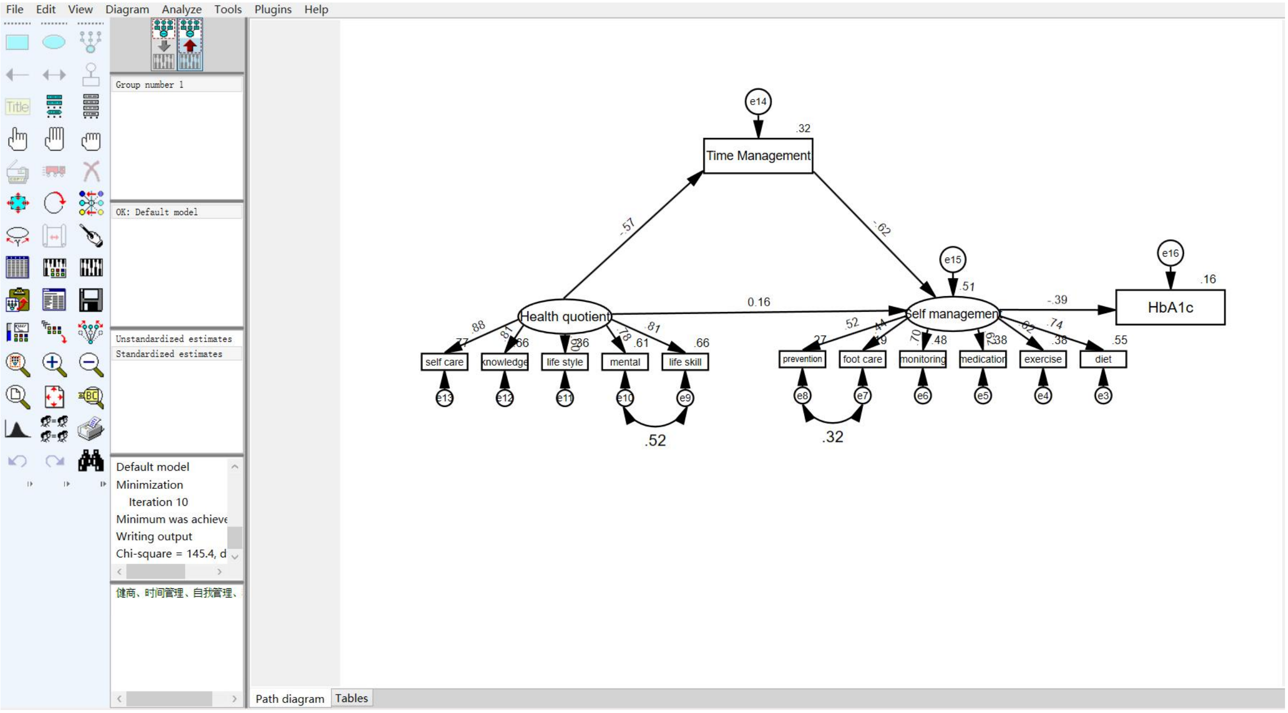

Analysis Summary
Notes for Group
Variable Summary
Parameter Summary
Assessment of normality
Observations farthest from the centroid (Mahalanobis distance)
Sample Moments
Notes for Model
Estimates
Modification Indices
Minimization History
Pairwise Parameter Comparisons
Summary of Bootstrap Iterations
Bootstrap Distributions
Model Fit
Execution Time

Estimates/Bootstrap
Estimates
Bootstrap standard errors
Bootstrap Confidence

Group number 1

Default model

Regression Weights: (Group number 1 - Default model)

			Estimate	S.E.	C.R.	P	Label
TimeManagement	<---	HealthQuotient	-11.424	1.371	-8.336	***	par_11
SelfManagement	<---	TimeManagement	-.343	.047	-7.286	***	par_12
SelfManagement	<---	HealthQuotient	1.728	.876	1.973	.048	par_13
diet	<---	SelfManagement	1.000				
exercise	<---	SelfManagement	1.430	.171	8.355	***	par_1
TakingMedication	<---	SelfManagement	.722	.087	8.256	***	par_2
GlucoseMonitoring	<---	SelfManagement	1.350	.152	8.898	***	par_3
FootCare	<---	SelfManagement	.477	.081	5.884	***	par_4
prevention	<---	SelfManagement	.891	.130	6.877	***	par_5
LifeSkill	<---	HealthQuotient	1.000				
MentalState	<---	HealthQuotient	.987	.054	18.129	***	par_6
LifeStyle	<---	HealthQuotient	.813	.089	9.098	***	par_7
HealthKnowledge	<---	HealthQuotient	1.542	.117	13.158	***	par_8
SelfCare	<---	HealthQuotient	1.189	.084	14.197	***	par_9
HbA1c	<---	SelfManagement	-.092	.018	-5.157	***	par_10

Standardized Regression Weights: (Group number 1 - Default model)

			Estimate
TimeManagement	<---	HealthQuotient	-.566
SelfManagement	<---	TimeManagement	-.618
SelfManagement	<---	HealthQuotient	.154
diet	<---	SelfManagement	.742
exercise	<---	SelfManagement	.619
TakingMedication	<---	SelfManagement	.618
GlucoseMonitoring	<---	SelfManagement	.695
FootCare	<---	SelfManagement	.441
prevention	<---	SelfManagement	.522
LifeSkill	<---	HealthQuotient	.813
MentalState	<---	HealthQuotient	.780
LifeStyle	<---	HealthQuotient	.603
HealthKnowledge	<---	HealthQuotient	.815
SelfCare	<---	HealthQuotient	.879
HbA1c	<---	SelfManagement	-.394

Covariances: (Group number 1 - Default model)

- Analysis Summary
- Notes for Group
- Variable Summary
- Parameter Summary
- Assessment of normality
- Observations farthest from the centroid (Mahalanobis distance)
- Sample Moments
- Notes for Model
- Estimates
- Modification Indices
- Minimization History
- Pairwise Parameter Comparisons
- Summary of Bootstrap Iterations
- Bootstrap Distributions
- Model Fit
- Execution Time

- Estimates/Bootstrap
  - Estimates
  - Bootstrap standard errors
  - Bootstrap Confidence

- Group number 1

- Default model

Total Effects (Group number 1 - Default model)

	HealthQuotient	TimeManagement	SelfManagement
TimeManagement	-11.424	.000	.000
SelfManagement	5.645	-.343	.000
HbA1c	-.517	.031	-.092
SelfCare	1.189	.000	.000
HealthKnowledge	1.542	.000	.000
LifeStyle	.813	.000	.000
MentalState	.987	.000	.000
LifeSkill	1.000	.000	.000
prevention	5.032	-.306	.891
FootCare	2.691	-.163	.477
GlucoseMonitoring	7.621	-.463	1.350
TakingMedication	4.077	-.248	.722
exercise	8.073	-.490	1.430
diet	5.645	-.343	1.000

Standardized Total Effects (Group number 1 - Default model)

	HealthQuotient	TimeManagement	SelfManagement
TimeManagement	-.566	.000	.000
SelfManagement	.504	-.618	.000
HbA1c	-.199	.244	-.394
SelfCare	.879	.000	.000
HealthKnowledge	.815	.000	.000
LifeStyle	.603	.000	.000
MentalState	.780	.000	.000
LifeSkill	.813	.000	.000
prevention	.263	-.323	.522
FootCare	.223	-.273	.441
GlucoseMonitoring	.351	-.430	.695
TakingMedication	.312	-.382	.618
exercise	.312	-.383	.619
diet	.374	-.459	.742

- Analysis Summary
- Notes for Group
- Variable Summary
- Parameter Summary
- Assessment of normality
- Observations farthest from the centroid (Mahalanobis distance)
- Sample Moments
- Notes for Model
- Estimates
- Modification Indices
- Minimization History
- Pairwise Parameter Comparisons
- Summary of Bootstrap Iterations
- Bootstrap Distributions
- Model Fit
- Execution Time

- Estimates/Bootstrap
  - Estimates
  - Bootstrap standard errors
  - Bootstrap Confidence

- Group number 1

- Default model

Direct Effects (Group number 1 - Default model)

	HealthQuotient	TimeManagement	SelfManagement
TimeManagement	-11.424	.000	.000
SelfManagement	1.728	-.343	.000
HbA1c	.000	.000	-.092
SelfCare	1.189	.000	.000
HealthKnowledge	1.542	.000	.000
LifeStyle	.813	.000	.000
MentalState	.987	.000	.000
LifeSkill	1.000	.000	.000
prevention	.000	.000	.891
FootCare	.000	.000	.477
GlucoseMonitoring	.000	.000	1.350
TakingMedication	.000	.000	.722
exercise	.000	.000	1.430
diet	.000	.000	1.000

Standardized Direct Effects (Group number 1 - Default model)

	HealthQuotient	TimeManagement	SelfManagement
TimeManagement	-.566	.000	.000
SelfManagement	.154	-.618	.000
HbA1c	.000	.000	-.394
SelfCare	.879	.000	.000
HealthKnowledge	.815	.000	.000
LifeStyle	.603	.000	.000
MentalState	.780	.000	.000
LifeSkill	.813	.000	.000
prevention	.000	.000	.522
FootCare	.000	.000	.441
GlucoseMonitoring	.000	.000	.695
TakingMedication	.000	.000	.618
exercise	.000	.000	.619
diet	.000	.000	.742

```
⊞ Analysis Summary
 Notes for Group
⊞ Variable Summary
 Parameter Summary
 Assessment of normality
 Observations farthest from the centroid (Mahalanobis distance)
⊞ Sample Moments
⊞ Notes for Model
⊞ Estimates
⊞ Modification Indices
 Minimization History
⊞ Pairwise Parameter Comparisons
 Summary of Bootstrap Iterations
⊞ Bootstrap Distributions
⊞ Model Fit
 Execution Time
```

```
⊟ Estimates/Bootstrap
 Estimates
 Bootstrap standard errors
⊞ Bootstrap Confidence
```

```
Group number 1
```

```
Default model
```

TakingMedication	.000	.000	.618
exercise	.000	.000	.619
diet	.000	.000	.742

Indirect Effects (Group number 1 - Default model)

	HealthQuotient	TimeManagement	SelfManagement
TimeManagement	.000	.000	.000
SelfManagement	3.917	.000	.000
HbA1c	-.517	.031	.000
SelfCare	.000	.000	.000
HealthKnowledge	.000	.000	.000
LifeStyle	.000	.000	.000
MentalState	.000	.000	.000
LifeSkill	.000	.000	.000
prevention	5.032	-.306	.000
FootCare	2.691	-.163	.000
GlucoseMonitoring	7.621	-.463	.000
TakingMedication	4.077	-.248	.000
exercise	8.073	-.490	.000
diet	5.645	-.343	.000

Standardized Indirect Effects (Group number 1 - Default model)

	HealthQuotient	TimeManagement	SelfManagement
TimeManagement	.000	.000	.000
SelfManagement	.350	.000	.000
HbA1c	-.199	.244	.000
SelfCare	.000	.000	.000
HealthKnowledge	.000	.000	.000
LifeStyle	.000	.000	.000
MentalState	.000	.000	.000
LifeSkill	.000	.000	.000
prevention	.263	-.323	.000
FootCare	.223	-.273	.000
GlucoseMonitoring	.351	-.430	.000
TakingMedication	.312	-.382	.000
exercise	.312	-.383	.000
diet	.374	-.459	.000
